# Circulating tumor DNA detection in lung cancer patients before and after surgery

**DOI:** 10.1038/srep33519

**Published:** 2016-09-19

**Authors:** Nannan Guo, Feng Lou, Yongfu Ma, Jie Li, Bo Yang, Wei Chen, Hua Ye, Jing-Bo Zhang, Ming-Yu Zhao, Wen-Jun Wu, Rong Shi, Lindsey Jones, Katherine S. Chen, Xue F. Huang, Si-Yi Chen, Yang Liu

**Affiliations:** 1 Medical School of Chinese PLA, Beijing, China; 2 Department of Cardiothoracic Surgery, the First Affiliated Hospital of General Hospital of the Chinese People’s Liberation Army, Beijing, China; 3 San Valley Biotechnology Incorporated, Beijing, China; 4 Department of Thoracic Surgery, The General Hospital of People’s Liberation Army, Beijing, China; 5 Norris Comprehensive Cancer Center, Department of Molecular Microbiology and Immunology, Keck School of Medicine, University of Southern California, Los Angeles, CA, USA

## Abstract

Circulating tumor DNA (ctDNA) in peripheral blood is a “liquid biopsy” that contains representative tumor information including gene mutations. Additionally, repeated ctDNA samples can be easily obtained to monitor response to treatment and disease progression, which may be especially valuable to lung cancer patients with tumors that cannot be easily biopsied or removed. To investigate the changes in ctDNA after surgical tumor resection, tumor and blood samples obtained before and after surgery were collected prospectively from 41 non-small lung cancer (NSCLC) patients. Somatic driver mutations in tumor DNA (tDNA) and pre- and post-op plasma ctDNA sample pairs were identified by targeted sequencing in several genes including *EGFR, KRAS*, and *TP53* with an overall study concordance of 78.1% and sensitivity and specificity of 69.2% and 93.3%, respectively. Importantly, the frequency of 91.7% of ctDNA mutations decreased after surgery and these changes were observed as little as 2 days post-op. Moreover, the presence of ctDNA had a higher positive predictive value than that of six tumor biomarkers in current clinical use. This study demonstrates the use of targeted sequencing to reliably identify ctDNA changes in response to treatment, indicating a potential utility of this approach in the clinical management of NSCLC.

Tumor characterization is the key to determining appropriate treatment for patients with cancer. This can be a particular challenge for some patients with non-small cell lung cancer (NSCLC), for lung tumor biopsies may be difficult if not impossible to obtain in many cases. Surgical resection offers the best hope of a cure, but this typically is not an option for those with advanced stage cancers. Because lung cancer is the most prevalent cancer worldwide^1^, continued efforts are focused on developing minimally invasive diagnostic techniques that can provide accurate diagnostic and prognostic information^2^.

Circulating tumor DNA (ctDNA) can be found in the peripheral blood of both early and late stage cancer patients, and has been found to contain gene mutations representative of those found in primary tumors^3^,^4^,^5^. These specific genetic changes found in ctDNA can have diagnostic value and predict responsiveness to treatment and patient survival^6^,^7^. Additionally, as these “liquid biopsies” are easily obtainable, repeated samples can be taken for real-time monitoring of both cancer patients’ response during the course of treatment and disease progression over time^8^,^9^. As such, peripheral blood liquid biopsies that contain tumor-representative ctDNA have been proposed as an adequate alternative to solid tumor biopsies^10^. Others have recently shown plasma ctDNA can be effectively used to monitor NSCLC patients during treatment, including for acquired drug resistance^11^,^12^. Unlike certain tumor protein biomarkers used to monitor disease progression that lack sensitivity and specificity^13^,^14^, plasma ctDNA concentrations have been shown to directly correlate to tumor burden, and additionally, it has been demonstrated that plasma ctDNA may be an independent prognostic biomarker for NSCLC, where patients with higher plasma ctDNA were shown to have poor responses to treatment and worse survival rates than those with lower plasma ctDNA concentrations^15^,^16^,^17^.

Generally, ctDNA represents a small fraction of the total plasma DNA, and as most ctDNA exists as 160–200 bp fragments^18^,^19^, sensitive detection methods are necessary. Recent studies have used various methods to detect genetic changes in ctDNA from lung cancer patients^4^,^9^,^20^. However, because there are still no clinical standard multiplex assays to simultaneously detect mutations across multiple genes in plasma ctDNA, additional research is warranted. Furthermore, the focus of most research on ctDNA mutations in lung cancer patients has been limited to a few genes that are frequently mutated in lung cancers with clinical implications, namely *EGFR* and *KRAS*^17^,^21^,^22^,^23^. Useful tools in the clinical characterization of plasma ctDNA from lung cancer patients may be the next-generation sequencing platforms as they have previously been demonstrated to have high sensitivity and can perform mutation screening in multiple genes simultaneously^24^,^25^,^26^,^27^. Additionally, these instruments and individual assays are relatively affordable with rapid run times^28^, making this targeted sequencing approach an attractive option for clinical use.

The aims of the present prospective study were to determine if a targeted sequencing approach could identify changes in plasma ctDNA mutation frequencies in blood samples obtained from NSCLC patients before and after surgical tumor resection, if the mutations identified in plasma ctDNA correspond to those in primary tDNA with high concordance, sensitivity, specificity, and positive predictive values (PPV), and if detecting plasma ctDNA is a more sensitive method than detecting certain clinically used tumor protein biomarkers.

## Results

**Figure 1:**
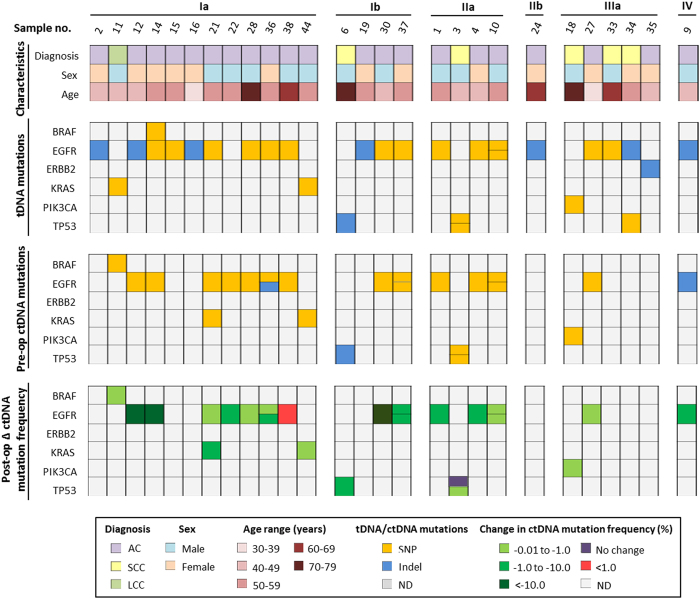
Summary of patient characteristics and gene mutations in matched tDNA and plasma ctDNA sample pairs. Patients were categorized based on stage, age, sex, and pathological diagnosis (top); type of mutation per gene in tDNA and pre-op plasma ctDNA (middle); and change in corresponding identified mutation frequency per gene in plasma ctDNA after surgery. tDNA and plasma ctDNA samples with two mutations in the same gene are indicated. Green indicates a decrease in mutation frequency, red indicates an increase in mutation frequency, white indicates no change in mutation frequency, and grey indicates no mutations were detected (ND) in that gene in tDNA (middle) or plasma ctDNA (bottom).

Prospective blood samples were taken from 41 NSCLC patients between 13 and 0 days prior to surgery (average 3.9 days) and again between 2 and 10 days after surgery (average 5.1 days), and isolated plasma ctDNA was compared to tDNA obtained during surgery. Sequencing analysis revealed that 27 of the 41 sample pairs (65.9%) contained one or more mutations in the 50 genes of the cancer panel in either or both sample types (Supplementary Table S1). Nine of the sample pairs contained two mutations in one or two genes. Altogether, 36 mutations were identified in tDNA and/or plasma ctDNA in *EGFR, KRAS, TP53, BRAF, PIK3CA*, and *ERBB2*. The average mutation frequency in tDNA was 20.0% (range: 1.38–83.7%). The majority (26/36; 72.2%) of all mutations were single nucleotide polymorphisms (SNPs), and the remaining mutations were inserts and deletions (Indels) (1/36 and 9/36, respectively). A summary of patient characteristics and associated tDNA mutations can be found in Fig. 1. DNA from white blood cells (WBC) from each patient was also analyzed and used for normalization to exclude the effect of germline mutations and ensure only somatic mutations were identified (Supplementary Table S1).

### Mutation concordance in matched tDNA and plasma ctDNA sample pairs

Of 41 matched tDNA and pre-op plasma ctDNA sample pairs, 18 contained concordant tDNA and plasma ctDNA mutations and 14 sample pairs contained no mutations in any of the genes screened in either tDNA or plasma ctDNA. Two different mutations were identified in each of five sample pairs: three sample pairs had concordant *EGFR* mutations in tDNA and plasma ctDNA but also an additional *EGFR* mutation found in either tDNA or plasma ctDNA only; one sample pair had a concordant *EGFR* mutation between sample types and a *TP53* mutation in plasma ctDNA only; and one sample pair contained a concordant *KRAS* mutation in both sample types and a discordant *BRAF* mutation found only in plasma ctDNA. Overall, the concordance rate of mutations identified in tDNA and matched pre-op plasma ctDNA was 78.1%, a sensitivity of 69.2% (95% CI 48.1–84.9%) and a specificity of 93.3% (95% CI 66.0–99.7%), with a PPV of cancer of 94.7% (95% CI 71.9–99.7%) (Supplementary Table S2).

For tDNA and plasma ctDNA from the 30 patients with earlier stage NSCLC (I and II), the mutation concordance rate was 80.0%, the sensitivity was 75.0% (95% CI 50.6–90.4%), the specificity was 90.0% (95% CI 54.1–99.5%), and the PPV was 93.8% (95% CI 67.7–99.7%) (Supplementary Table S3). Eleven samples from more advanced stage cancer patients (III and IV), on the other hand, had a mutation concordance of 72.7%, a sensitivity of 50.0% (95% CI 14.0–86.1%), a specificity of 100.0% (95% CI 46.3–100.0%), and a PPV of 100.0% (95% CI 40.0–100.0%) (Supplementary Table S4). The increased specificity and PPV in samples from late stage NSCLC patients compared to those from early stage patients may be reflective of the increased plasma ctDNA levels found as tumor burden increases.

### Plasma ctDNA mutation frequencies before and after surgery

**Figure 2:**
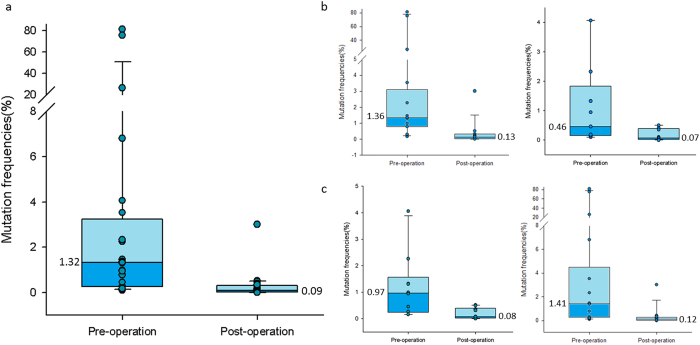
(**a**) Box-and-whisker plot of the mutation frequencies of the 24 somatic mutations detected in plasma ctDNA from all patients pre- and post-operation. (**b**) Change in plasma ctDNA mutation frequencies after surgery for 15 mutations found in 12 stage Ia/Ib patients (left) versus that in 9 mutations in 7 stage IIa-IV patients (right). (**c**) Change in plasma ctDNA mutation frequencies in 10 blood samples taken 2–4 days post-op (left) versus 14 blood samples taken 5–8 days post-op (right).

Overall, 19 plasma samples (46.3%) were positive for ctDNA before surgery, and among these 19 samples a total of 24 mutations were found in *EGFR* (17/24; 70.8%), *KRAS* (2/24; 8.3%), *TP53* (3/24; 12.5%), *BRAF* (1/24; 4.2%), and *PIK3CA* (1/24; 4.2%). Across all genes with mutations, the average plasma ctDNA mutation frequency before surgery was 8.88% (range: 0.1–81.06%) whereas the average post-surgery plasma ctDNA mutation frequency was dramatically reduced to 0.28% (range: 0.00–3.01%). Twenty-two (91.7%) of the identified plasma ctDNA mutations had a decrease in mutation frequency before and after surgery (avg. 9.46%, range: 0.08–80.74%), one of the plasma ctDNA mutations had no change in frequency, and one mutation showed an increase in mutation frequency (1.55%) (Fig. 1). Figure 2a illustrates the decreases in the 24 identified plasma ctDNA mutation frequencies after surgical tumor resection compared to those found in plasma ctDNA samples taken prior to surgery, where the median mutation frequency of pre-op versus post-op samples is 1.32% and 0.09%, respectively. The mutation frequency in plasma ctDNA from stage Ia and Ib cancer patients had the most dramatic decrease after surgery (avg. 11.52% and 14.63%, respectively), whereas the mutation frequency in plasma ctDNA from more advanced cancer patients (stages IIa and IIIa) decreased to a lesser degree (avg. 0.57% and avg. 0.13%, respectively). The median change in plasma ctDNA mutation frequency after surgery comparing early stage cancers versus more advanced cancers is shown in Fig. 2b. The plasma ctDNA mutation frequency from the one stage IV patient decreased from 4.06% pre-op to 0.00% post op.

All patient blood samples were taken between 2 and 10 days post-surgical tumor resection, and the 24 plasma ctDNA mutations identified were from blood samples taken between 2 and 8 days after surgery. Figure 2c illustrates that there is no significant difference between median plasma ctDNA mutation frequency from 10 samples taken 2–4 days post-op and that from 14 samples taken 5–8 days post-op (0.08% vs. 0.12%, respectively. Additionally, the total amount of cell-free DNA (cfDNA) isolated from patient plasma samples obtained before and after surgery was quantified (Supplementary Table S5). In most patients (30/41; 73.2%), the amount of cfDNA increased post-op by between 0.02 and 1.51 ng/μl (avg. 0.329 ± 0.333 ng/μl), whereas cfDNA decreased in 11 patients after surgery by between 0.01 and 0.122 ng/μl (avg. 0.063 ± 0.037 ng/μl).

### Plasma ctDNA detection versus tumor biomarkers

**Figure 3:**
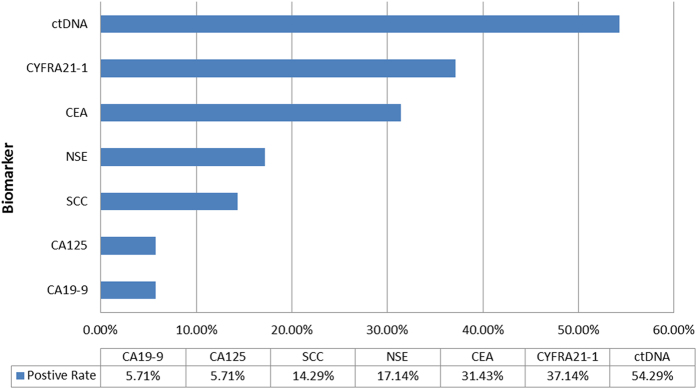
Positive detection rates of plasma ctDNA versus tumor biomarkers (n = 35 patients positive for one or more tumor biomarkers).

In addition to ctDNA, pre-surgery plasma samples were analyzed for the presence of the following six tumor biomarkers: CA125, CA19-9, CYFRA21-1, CEA, NSE, and squamous cell carcinoma antigen (SCC) (Supplementary Table S6). Overall, 34 plasma samples were positive for one or more of the biomarkers analyzed, where 13 were positive for CYFRA21-1, six were positive for NSE and CEA each, five were positive for SCC, and CA19-9 and CA125 were both positive in two samples (Fig. 3). In contrast, 18 of these 34 plasma samples were positive by ctDNA detection. Thus plasma ctDNA had a higher detection rate and higher PPV compared to these six tumor biomarkers.

## Discussion

The present prospective study examined the use of targeted sequencing to monitor the change in mutation frequency in plasma ctDNA from NSCLC patients before and after surgical lung tumor resection. Analysis of the tDNA revealed one or more mutations in 27 tumor samples. Eighteen tDNA and matched plasma ctDNA samples obtained before and after surgery contained mutations in *EGFR, KRAS, TP53, BRAF, PIK3CA*, and *ERBB2*. Mutations were not detected in any of the 50 genes screened in either the tDNA or plasma ctDNA of 14 samples pairs. The overall study concordance in tDNA and plasma ctDNA mutations was 78.1%, with a sensitivity of 69.2% and specificity of 93.3%. The high PPV of 94.7% indicates the accuracy of the test and suggests that the presence of plasma ctDNA may be a good indicator of cancer.

Of the 24 mutations identified in pre-op plasma ctDNA, 91.7% (22/24) decreased in frequency after surgery, and this decrease could be observed as little as 2 days post-op. Interestingly, in the majority of patients (30/41), the concentration of cell-free DNA (cfDNA) increased after surgery, which may have been the result of surgical wound healing causing cfDNA fragments from healthy cells to be released into the blood. Of the patients with ctDNA mutations identified with decreased variant frequencies after surgery, five also had a decrease in post-op cfDNA concentration whereas 12 had an increase in post-op cfDNA concentration, indicating that the variant frequency was not influenced by the total amount of cfDNA in this study.

Some mutations were identified in plasma ctDNA but not in tDNA. One might expect to find this in patients with metastatic disease, where the ctDNA possibly originated from a secondary tumor which may have a different mutation profile. However, the mutations identified in ctDNA only were in patients with early stage disease. This may be due to intratumoral heterogeneity characteristic of lung cancer^29^, as only portions of the tumors were used for sequencing analysis. While we considered these mutations found in plasma ctDNA only to be false positives for statistical analysis, these mutations could indeed be from a portion of the primary tumor or a metastatic site that were not analyzed in our study. Similarly, while mutations found in tDNA but not plasma ctDNA were considered to be false negatives, these mutations may have been present in circulation but at levels insufficient to detect by the Ion Proton, as the detection limit was determined to be 0.1% variant frequency and ctDNA accounts for only 0.02% to 0.1% of all circulating DNA assayed^3^. A challenge in ctDNA mutation monitoring lies in the detection threshold where *in vivo* levels may be below detectable limits. A disadvantage of targeted sequencing of ctDNA is that this method cannot determine if the mutations originate from primary or metastatic tumors; however, because ctDNA levels correlate with tumor burden, an increase in ctDNA may be indicative of disease recurrence or progression.

While surgery offers the best possibility of a cure for early stage NSCLC patients it is not an option for many patients, particularly those with late stage or metastatic disease. For these patients, monitoring plasma ctDNA can provide information on disease progression, as ctDNA levels have been shown to be directly correlated with tumor size and stage^5^,^17^,^30^. A useful, non-invasive, and convenient way to monitor cancer progression over time would also be to measure tumor biomarkers such as CEA, SCC, and CYFRA21-1 in patient serum. However, such biomarkers alone are not enough to diagnose or monitor lung cancer because they lack sensitivity and specificity to a single cancer type or may be elevated due to unrelated issues^31^,^32^. Plasma ctDNA offers more insight into the specific disease condition, as the ctDNA originates from tumors and contains mutations only present in tumor cell DNA and thus serves as an extremely specific cancer biomarker that can be detected and monitored over time^10^.

Mutations found in plasma ctDNA can also provide useful information for targeted therapies. For example, erlotinib is a tyrosine kinase inhibitor (TKI) approved for first-line treatment of metastatic NSCLC in patients with *EGFR* exon 19 deletions or exon 21 (L858R) substitution mutations^33^. However, approximately half of all patients receiving erlotinib or other EGFR inhibitors acquire resistance from a secondary mutation in *EGFR* (T790M) causing the drugs to lose their effectiveness^34^. A targeted sequencing platform such as the Ion Proton with a defined panel of genes could identify both the primary mutation before treatment and rapidly detect the secondary mutation to indicate that treatment such as erlotinib should be stopped or switched to a more effective third-generation TKI. Thus plasma ctDNA mutation monitoring may be especially important for patients who will receive anti-EGFR therapy.

In conclusion, the present study demonstrated that the pre-op and post-op changes in plasma ctDNA mutation frequencies can be detected through targeted sequencing with the Ion Proton. While the ctDNA mutations in plasma samples obtained before surgery had a high concordance rate to mutations found in primary tumor tissue and greater PPV than the detection of tumor marker expression, these results need to be further validated in larger studies and matched with clinical outcome data in order to fully demonstrate the utility of this targeted sequencing method in a diagnostic setting and in adjuvant chemotherapy selection. Based on the limited results herein, the rapid detection of plasma ctDNA and mutations with targeted sequencing has potential to be a useful clinical strategy to monitor patients’ response to treatment, including surgery.

## Methods

### Ethics Statement and Patients

The study has been approved by the General Hospital of the People’s Liberation Army Ethics Committee and the methods were carried out in accordance with the approved guidelines. All patients provided appropriate written informed consent for the use of blood and lung tumor tissue under the approval of the Ethics Committee. All samples and medical data used in this study have been irreversibly anonymized. NSCLC patients who underwent surgical treatments in the Department of Thoracic Surgery at the General Hospital of the People’s Liberation Army were enrolled in this study, including 22 males and 19 females, aged 38–73 years (median age of 52 years) (Table 1). Blood samples from each patient were obtained between 13 and 0 days prior to surgery (avg. 3.9 days) and 2–10 days after surgery (avg. 5.1 days).

### Tumor Tissue and Blood Sample DNA Preparation

To process lung tumor samples, the E.Z.N.A. Tissue DNA kit (Omega Bio-Tek, Norcross, GA) was used for DNA extraction from sections of fresh tumor tissue confirmed to contain greater than 30% tumor cells, and the QIAamp DNA FFPE Tissue kit (QIAGEN, Valencia, CA) was used to extract DNA from 3–5 μm thick sections of formalin-fixed, paraffin-embedded (FFPE) tissue, following the manufacturer’s respective instructions. EDTA tubes containing patient blood samples were centrifuged at 1600 g or 10 min and the peripheral blood lymphocyte (PBL) debris was then stored at −20 °C until use. The supernatants from these samples were further centrifuged for 10 min at 16,000 g, and plasma was collected and stored at −80 °C until needed. DNA was extracted from the PBLs using the E.Z.N.A. Blood DNA kit (Omega Bio-Tek) and ctDNA was extracted from at least 1 ml plasma using the QIAamp Circulating Nucleic Acid kit (QIAGEN) as per the manufacturers’ respective instructions. Finally, DNA was quantified with the Qubit 2.0 Fluorometer and Qubit dsDNA HS Assay kit (Life Technologies, Carlsbad, CA) according to the recommended protocol.

### Ion Proton Library Preparation and Sequencing

Preparation of the Ion Proton library and DNA sequencing was performed as described in our previous publications^35^,^36^,^37^. For each sample type, an adapter-ligated library was generated with the Ion AmpliSeq Library Kit 2.0 (Life Technologies) according to the manufacturer’s protocol. Briefly, 10∼20 ng of pooled amplicons were end-repaired and ligated to Ion Adapters X and P1. AMPure beads (Beckman Coulter, Brea, CA) were used to purify adapter-ligated products, followed by nick-translation and PCR-amplification for a total of 5 cycles. AMPure beads were used to purify the resulting library, and the Agilent 2100 Bioanalyzer and Agilent Bioanalyzer DNA High-Sensitivity LabChip (Agilent Technologies) were used to determine the concentration and size of the library. Sample emulsion PCR and emulsion breaking were performed using the Ion OneTouch system (Life Technologies) with the Ion PI Template OT2 200 Kit v3 (Life Technologies) as per the manufacturer’s instructions. Ion Sphere Particles (ISPs) were recovered, and template-positive ISPs were enriched with Dynabeads MyOne Streptavidin C1 beads (Life Technologies) on the Ion One Touch ES (enrichment system) (Life Technologies), and ISP enrichment was confirmed using the Qubit 2.0 Fluorometer (Life Technologies). The Ion Proton System using Ion PI v2 Chips (Life Technologies) were used to sequence barcoded samples for 100 cycles and the Ion PI Sequencing 200 Kit v3 (Life Technologies) was used for sequencing reactions following the recommended protocol.

We used the SV-CA50-ctDNA panel (San Valley Biotech Inc., Beijing, China) that is capable of detecting somatic mutations from plasma or tissue samples in the following 50 cancer related genes: *ABL1, AKT1, ALK, APC, ATM, BRAF, CDH1, CDKN2A, CSF1R, CTNNB1, EGFR, ERBB2, ERBB4, EZH2, FBXW7, FGFR1, FGFR2, FGFR3, FLT3, GNA11, GNAQ, GNAS, HNF1A, HRAS, IDH1, IDH2, JAK2, JAK3, KDR, KIT, KRAS, MET, MLH1, MPL, NOTCH1, NPM1, NRAS, PDGFRA, PIK3CA, PTEN, PTPN11, RB1, RET, SMAD4, SMARCB1, SMO, SRC, STK11, TP53*, and *VHL*. These genes and their 2856 mutational hotspot loci were selected based on the Chinese cancer sequence database (San Valley Inc.) that contains sequencing data from more than 30,000 cancer patients and more than 13 kinds of cancers. Of these 30,000 tumor samples in the database, over 20% are lung cancer, 19% are gastric cancer, 14% are colorectal cancer, and 10% are breast cancer, with additional data from liver cancer, esophageal cancer, and gastric stromal tumors. Since the ctDNA in plasma is comprised of short DNA fragments, amplicons in the panel are specially-designed for efficient amplification of ctDNA.

For plasma samples, the total reads were all more than 2.07 million to ensure the average base coverage depth was over 10,000x. Additionally, the average uniformity of base coverage was 95.5%. Theses strict quality control criteria ensured the reliability of sequencing.

### Variant Calling

To determine the minimum variant frequency threshold, A plasmid with 100% *EGFR* p.L858R and *EGFR* p.E746_A750delELREA mutations was used as a positive control, and the negative control was a wild type plasmid at these positions. HindIII enzyme sites were inserted into both plasmids allowing the plasmids to be divided into 180 bp DNA segments. These two plasmids were mixed in 0.0%, 0.1%, 0.5%, 1.0% proportions of mutation reference standards. Each proportion of reference standard was sequenced 10 times using the 50 genes cancer panel with 10,000x sequencing depth. The results indicated that the detection limit of our sequencing method is 0.1%.

Initial data from the sequencing runs was processed with the Ion Proton platform-specific pipeline software Torrent Suite to generate sequence reads, trim adapter sequences, and filter and remove poor signal-profile reads as described in our previous publications^35^,^36^,^37^. Initial variant calling from the sequencing data was generated with the Torrent Suite Software with a plug-in “variant caller v4.0”. Three filtering steps were used to eliminate erroneous base calling and generate final variant calling. For the first filter, the following were defined for tDNA: the average total coverage depth > 1000; each variant coverage > 20; a variant frequency of each sample > 5%; and *p* value < 0.01; and the following were defined for plasma ctDNA: the average total coverage depth > 10000; each variant coverage > 10; a variant frequency of each sample > 0.1%; and *p* value < 0.01. The second filtering step utilized the Integrative Genomics Viewer (IGV) software (http//www.broadinstitute.org/igv) or Samtools software (http://samtools.sourceforge.net) to eliminate possible DNA strand-specific errors after visual examination of called mutations. The final filter set variants within the 739 mutational hotspots, according to the manufacturer’s instructions.

### Tumor Protein Biomarker Analysis

In addition to ctDNA mutation analysis, pre-surgery plasma samples were analyzed for the presence of the following tumor biomarkers: cancer antigen 125 (CA125), carbohydrate antigen 19-9 (CA19-9), carcinoembryonic antigen (CEA), cytokeratin 19 fragment (CYFRA21-1), and neuron specific enolase (NSE). Levels of serum CA125, CA19-9, CYFRA21-1, CEA, SCC, and NSE were measured with standard RIA, chromatometrychemoluminescence, and ELISA methods, respectively.

### Statistical Analysis

For statistical analysis, tDNA was used as the reference when compared to plasma ctDNA. Matched tDNA and plasma ctDNA samples with the same mutations were considered to be true positives, and matched sample pairs without mutations (wild type) in the 50 genes screened were considered to be true negatives. Sample pairs with mutations identified in plasma ctDNA but not tDNA were considered to be false positives, whereas sample pairs with mutations found in tDNA but not plasma ctDNA were considered to be false negatives. Sensitivity, specificity, and concordance rate ([(true positive þ true negative)/n]) were calculated. Plasma predictive value was calculated as the number of true positives plus false positives divided by the total number of samples. Analyses were performed using the SPSS Statistics version 19 (IBM Corp).

## Additional Information

**How to cite this article**: Guo, N. *et al*. Circulating tumor DNA detection in lung cancer patients before and after surgery. *Sci. Rep.* **6**, 33519; doi: 10.1038/srep33519 (2016).

## Supplementary Material

Supplementary Information

## Figures and Tables

**Table 1:** Clinical features of 41 NSCLC cancer patients.

Characteristic	Parameter value
Age, years	
Mean (SD)	55 (10.38)
Median (range)	52 (38–73)
Sex	
Male	22
Female	19
Non-small cell lung cancer subtype	41
adenocarcinoma	33
squamous cell carcinoma	6
neuroendocrine carcinoma	1
large cell carcinoma	1
Tumor stage	
Ia	17
Ib	6
IIa	6
IIb	1
IIIa	10
IV	1
