# Supplementary material for: Circulating tumor DNA detection in lung cancer patients before and after surgery
Source: Sci Rep. 2016 Sep 19;6:33519. doi: 10.1038/srep33519 (PMC5027588; doi:10.1038/srep33519)
Supplement: Supplementary Information [file srep33519-s1.pdf]

Circulating tumor DNA detection in lung cancer patients before and after surgery

Nannan Guo[#], Feng Lou[#], Yongfu Ma[#], Jie Li, Bo Yang, Wei Chen, Hua Ye, Jing-Bo Zhang, Ming-Yu Zhao, Wen-Jun Wu, Rong Shi, Lindsey Jones, Katherine S Chen, Xue F Huang, Si-Yi Chen[*], and Yang Liu[*]

Supplementary Table S1. Mutations and mutation frequencies identified in tDNA and plasma ctDNA before and after surgery.

Patient no.	Gene	Mutation type	AA mutation	% mutation in tDNA (reads)	% mutation in pre-op ctDNA (reads)	% mutation in post-op ctDNA (reads)	% mutation in WBC (reads)	Detected in tDNA/ctDNA
1	EGFR	SNP	p.L858R	4.00 (23606)	2.33 (17676)	0.41 (15955)	0.00 (19588)	Y/Y
2	EGFR	DEL	p.K745_E749del	11.17 (20207)	0.00 (27639)	0.00 (22599)	0.00 (13271)	Y/N
3	TP53	SNP	p.E204*	34.84 (11212)	0.10 (37966)	0.10 (12839)	0.00 (20164)	Y/Y
	TP53	SNP	p.V203V	35.04 (11255)	0.15 (37975)	0.06 (12840)	0.00 (20138)	Y/Y
4	EGFR	SNP	p.L858R	19.54 (9970)	1.33 (21980)	0.02 (4975)	0.00 (7505)	Y/Y
6	TP53	DEL	p.S303fs*42	17.47 (3681)	6.80 (9809)	0.00 (4987)	0.00 (2047)	Y/Y
9	EGFR	DEL	p.E746_A750delELREA	13.46 (9685)	4.06 (9997)	0.00 (10000)	0.00 (9799)	Y/Y
10	EGFR	SNP	p.L858R	4.44 (17785)	0.94 (28900)	0.50 (12618)	0.00 (9527)	Y/Y
	EGFR	SNP	p.G810D	4.32 (14110)	0.16 (65062)	0.07 (37789)	0.00 (8381)	Y/Y
11	KRAS	SNP	p.G13C	19.64 (560)	0.01 (15793)	0.01 (20036)	0.00 (851)	Y/N
	BRAF	SNP	p.S605G	0.00 (189)	0.99 (15824)	0.32 (13517)	0.00 (219)	N/Y
12	EGFR	DEL	p.E746_A750delELREA	6.63 (7149)	0.00 (22096)	0.00 (18587)	0.00 (2460)	Y/N
	EGFR	SNP	p.L858R	0.06 (16602)	26.44 (16791)	0.02 (22712)	0.00 (3463)	N/Y
14	BRAF	SNP	p.V600E	1.38 (799)	0.01 (7174)	0.01 (11450)	0.00 (619)	Y/N
	EGFR	SNP	p.L858R	6.34 (6909)	81.06 (4689)	0.33 (23855)	0.00 (2548)	Y/Y
15	EGFR	SNP	p.S768I	7.82 (307)	0.01 (15966)	0.00 (2236)	0.00 (351)	Y/N
16	EGFR	DEL	p.L747_P753>S	15.48 (1169)	0.00 (51613)	0.00 (21521)	0.00 (1180)	Y/N
18	PIK3CA	SNP	p.K111R	9.16 (764)	0.46 (7671)	0.36 (7291)	0.00 (280)	Y/Y
19	EGFR	DEL	p.E746_A750delELREA	26.02 (2563)	0.00 (26959)	0.00 (11927)	0.00 (6964)	Y/N
21	KRAS	SNP	p.G12D	0.16 (1256)	2.26 (19251)	0.15 (15248)	0.00 (1569)	N/Y
	EGFR	SNP	p.L858R	10.26 (10608)	0.16 (18354)	0.05 (14744)	0.00 (5944)	Y/Y
22	EGFR	SNP	p.L858R	0.10 (4919)	1.31 (15007)	0.08 (18930)	0.00 (5398)	N/Y
24	EGFR	DEL	p.L747_P753>S	17.87 (414)	0.00 (21464)	0.00 (22992)	0.00 (11647)	Y/N
27	EGFR	SNP	p.L858R	30.06 (6663)	0.19 (14512)	0.02 (11352)	0.00 (6387)	Y/Y
28	EGFR	SNP	p.L858R	29.67 (6930)	0.29 (14815)	0.01 (14138)	0.00 (10015)	Y/Y
30	EGFR	SNP	p.L858R	83.71 (23376)	75.45 (81301)	0.13 (11140)	0.00 (6041)	Y/Y
33	EGFR	SNP	p.L858R	2.44 (4018)	0.035 (22648)	0.036 (18954)	0.00 (9395)	Y/N
34	EGFR	DEL	p.L747_P753>S	73.26 (25063)	0.04 (28813)	0.04 (21551)	0.00 (3241)	Y/N
	TP53	SNP	p.C238F	34.95 (8931)	0.00 (57159)	0.01 (59452)	0.00 (7188)	Y/N
35	ERBB2	INS	p.A775_G776insYVMA	54.7 (8931)	0.00 (57159)	0.00 (59452)	0.00 (5756)	Y/N
36	EGFR	SNP	p.L858R	16.92 (4220)	0.78 (17892)	0.14 (13248)	0.00 (11387)	Y/Y
	EGFR	DEL	p.E746_A750delELREA	0.00 (3164)	3.53 (25172)	0.06 (24795)	0.00 (8245)	N/Y
37	EGFR	SNP	p.L858R	25.39 (2225)	1.36 (15867)	0.24 (15465)	0.00 (2847)	Y/Y
	EGFR	SNP	p.L861Q	0.00 (2242)	1.34 (15918)	0.19 (15554)	0.00 (2825)	N/Y
38	EGFR	SNP	p.L858R	2.60 (5452)	1.46 (16551)	3.01 (11418)	0.00 (5505)	Y/Y
44	KRAS	SNP	p.G13D	20.71 (1811)	0.26 (25839)	0.02 (13372)	0.00 (1735)	Y/Y

SNP: single nucleotide polymorphism; DEL: deletion; INS: insert; WBC: white blood cells

Supplementary Table S2. Concordance, specificity, sensitivity, and positive predictive value calculations for all samples in the study.

	**Plasma mutation status**		
	Positive	Negative	**Total**
**Tumor mutation status**			
Positive	18	8	26
Negative	1	14	15
**Total**	19	22	41

	**n**	**Rate (%)**	**95% Confidence interval (%)**	
Concordance	41	78.05		
Sensitivity	26	69.23	48.10	84.91
Specificity	15	93.33	66.03	99.65
Positive-predictive value	19	94.74	71.89	99.72

Supplementary Table S3. Concordance, specificity, sensitivity, and positive predictive value calculations for early stage (I-II) samples in the study.

	**Plasma mutation status**		
	Positive	Negative	**Total**
**Tumor mutation status**			
Positive	15	5	20
Negative	1	9	10
**Total**	16	14	30

	**n**	**Rate (%)**	**95% Confidence interval (%)**	
Concordance	30	80.00		
Sensitivity	20	75.00	50.59	90.41
Specificity	10	90.00	54.12	99.48
Positive-predictive value	16	93.75	67.71	99.67

Supplementary Table S4. Concordance, specificity, sensitivity, and positive predictive value calculations for late stage (III-IV) samples in the study.

	**Plasma mutation status**		
	Positive	Negative	**Total**
**Tumor mutation status**			
Positive	3	3	6
Negative	0	5	5
**Total**	3	8	11

	**n**	**Rate (%)**	**95% Confidence interval (%)**	
Concordance	11	72.73		
Sensitivity	6	50.00	13.95	86.05
Specificity	5	100.00	46.29	100.00
Positive-predictive value	3	100.00	40.00	100.00

Supplementary Table S5. Patient cfDNA concentrations before and after surgery (ng/µl).

Patient no.	Pre-op cfDNA (ng/µl)	Post-op cfDNA (ng/µl)	Change in cfDNA concentration (+/-)
1	0.964	1.07	+0.106
2	1.39	1.45	+0.06
3	0.848	1.36	+0.512
4	0.50	1.16	+0.66
5	0.464	0.738	+0.274
6	0.952	1.06	+0.108
7	1.04	0.994	-0.046
9	0.694	1.52	+0.826
10	0.968	1.06	+0.092
11	0.688	0.618	-0.07
12	0.664	0.602	-0.062
14	0.702	0.58	-0.122
15	0.636	0.796	+0.16
16	0.624	0.592	-0.032
17	0.428	0.654	+0.226
18	0.542	0.746	+0.204
19	0.478	1.02	+0.542
20	0.344	0.40	+0.056
21	0.516	0.853	+0.337
22	0.424	0.388	-0.036
23	0.444	0.91	+0.466
24	0.908	0.978	+0.07
25	0.79	1.25	+0.46
26	0.564	0.904	+0.34
27	0.442	0.618	+0.176
28	0.516	0.79	+0.274
29	0.34	1.85	+1.51
30	0.422	0.558	+0.136
31	0.492	0.686	+0.194
33	0.406	0.396	-0.01
34	0.36	0.38	+0.02
35	0.40	0.64	+0.24
36	0.334	0.41	+0.076
37	0.294	0.594	+0.30
38	0.478	0.386	-0.092
39	0.104	0.364	+0.26
40	0.49	1.57	+1.08
41	0.276	0.256	-0.02
42	0.284	0.176	-0.108
44	0.23	0.14	-0.09
45	0.14	0.246	+0.106

Supplementary Table S6. Tumor biomarker levels of patients. The positive cut-off values were defined as follows: CA125 >35 U/ml, CA19-9 >39 U/ml, CEA >4.7 ng/ml, CYFRA21-1 >3.3ng/ml, NSE >16.3 ng/ml, and SCC >1.5 ng/ml. (ND: not detected)

Patient no.	CEA (μg/L)	CA125 (U/mL)	CA19-9 (U/mL)	CYFRA21-1 (ng/ml)	NSE (ng/ml)	SCC (ng/ml)
1	16.67	26.69	65.79	4.40	11.04	0.70
2	ND	ND	ND	ND	ND	ND
3	2.58	ND	ND	3.16	12.34	0.70
4	10.59	11.46	7.39	2.15	9.35	0.90
5	16.57	18.33	8.02	2.47	12.5	0.60
6	95.98	11.93	13.36	19.33	26.0	10.60
7	3.76	4.77	6.39	4.54	10.45	0.60
9	4.39	25.84	9.8	2.08	13.03	1.00
10	28.68	10.1	11.05	4.34	11.56	1.10
11	6.3	14.46	7.32	1.02	13.78	1.10
12	2.62	29.61	16.9	0.94	11.19	1.10
14	3.35	19.51	1.00	1.65	13.62	0.80
15	3.91	12.44	18.52	1.80	9.81	0.90
16	ND	ND	ND	ND	ND	ND
17	3.45	9.51	8.07	1.62	6.75	0.70
18	3.77	24.09	9.92	11.91	26.77	1.10
19	3.95	12.23	4.61	2.31	12.44	0.80
20	1.78	3.17	7.80	2.63	12.78	0.90
21	2.49	7.96	7.26	2.09	8.93	0.60
22	0.82	15.34	18.87	2.62	12.27	0.60
23	2.11	10.87	14.22	1.39	14.78	0.60
24	40.7	164.9	29.61	6.33	11.44	0.90
25	2.46	9.94	11.83	1.96	15.96	0.70
26	8.51	5.08	5.05	2.11	16.00	0.40
27	1.78	20.41	8.71	1.77	9.06	0.40
28	ND	ND	ND	ND	ND	ND
29	ND	ND	ND	ND	ND	ND
30	154.9	26.94	16.13	11.55	16.49	0.60
31	ND	ND	ND	ND	ND	ND
33	7.04	19.33	8.41	118.7	26.11	4.50
34	1.28	5.26	10.2	3.75	13.71	0.60
35	18.56	12.8	11.07	10.25	14.79	1.20
36	ND	ND	ND	ND	ND	ND
37	4.35	8.14	4.32	2.03	10.45	0.40
38	1.29	6.00	7.19	1.23	9.13	0.60
39	2.02	11.23	16.18	3.07	7.65	3.50
40	3.51	26.66	42.91	2.38	16.42	0.90
41	4.55	174.5	24.4	25.79	19.81	1.90
42	0.76	13.16	5.34	2.13	11.84	1.1
44	2.00	4.53	7.52	3.32	8.19	1.4
45	3.49	20.29	10.04	4.62	10.89	3.5
